# Spider Silk as Guiding Biomaterial for Human Model Neurons

**DOI:** 10.1155/2014/906819

**Published:** 2014-05-18

**Authors:** Frank Roloff, Sarah Strauß, Peter M. Vogt, Gerd Bicker, Christine Radtke

**Affiliations:** ^1^Division of Cell Biology, University of Veterinary Medicine Hannover, Bischofsholer Damm15/102, 30173 Hannover, Germany; ^2^Department of Plastic, Hand and Reconstructive Surgery, Hannover Medical School, Carl-Neuberg-Straße 1, 30625 Hannover, Germany; ^3^Center for Systems Neuroscience, Hannover, Germany

## Abstract

Over the last years, a number of therapeutic strategies have emerged to promote axonal regeneration. An attractive strategy is the implantation of biodegradable and nonimmunogenic artificial scaffolds into injured peripheral nerves. In previous studies, transplantation of decellularized veins filled with spider silk for bridging critical size nerve defects resulted in axonal regeneration and remyelination by invading endogenous Schwann cells. Detailed interaction of elongating neurons and the spider silk as guidance material is unknown. To visualize direct cellular interactions between spider silk and neurons *in vitro*, we developed an *in vitro* crossed silk fiber array. Here, we describe in detail for the first time that human (NT2) model neurons attach to silk scaffolds. Extending neurites can bridge gaps between single silk fibers and elongate afterwards on the neighboring fiber. Culturing human neurons on the silk arrays led to an increasing migration and adhesion of neuronal cell bodies to the spider silk fibers. Within three to four weeks, clustered somata and extending neurites formed ganglion-like cell structures. Microscopic imaging of human neurons on the crossed fiber arrays *in vitro* will allow for a more efficient development of methods to maximize cell adhesion and neurite growth on spider silk prior to transplantation studies.

## 1. Introduction

The development of artificial nerve conduits as guiding channels for regenerating axons is of increasing interest as an alternative to autologous nerve grafts which have limitations of amount and size of tissue and potential donor site morbidity [1, 2]. The principle in tubular nerve guidance channels is that nerve stumps of the transected nerves on both sides are inserted and therefore act as growth support for the regenerating nerve fibers from the proximal to the distal nerve stumps. As guidance channels various materials can be used either made out of natural biological tissue, for example, autologous vein, arteries, and skeletal muscle, or synthetic materials including silicone or biodegradable polymers like polyglycolic acid (PGA), poly L-lactic acid (PLLA), poly-3-hydroxybutyrate (PHB), and their copolymers or derivatives [3–5]. As a first step to successful scaffold implantation, the conduit material has to be properly selected: most desirable is a biodegradable conduit because permanent tubes can lead to foreign body reaction and to inhibition of nerve growth by excessive scar tissue formation. Moreover, the nerve conduit should not induce an immunological response or be toxic. In previous studies we demonstrated that the artificial nerve grafts consisting of decellularized veins filled with spider silk could be used to bridge long-distance defects of peripheral nerves in adult sheep [6]. The regenerated axons were found 8 months after lesion induction and nerve repair, and moreover, the axons were as well myelinated by endogenous invaded Schwann cells. The most significant limiting factor for nerve defect regeneration is the gap length which has to be bridged. Most available conduits support substance defects up to 30 mm and are designed for repairing small-diameter nerves. To further improve results, nerve tubes have been modified to contain a supporting structure to stabilize the conduit, filled with collagen and laminin-containing gels, or combined with growth factors or cells to enhance regeneration. It will be important in future work to determine if constructs seeded with cells enhance nerve regeneration and functional outcome as compared to scaffold material alone. So far these nerve conduits have been successfully applied in transplantation studies in the peripheral nervous system of a large animal model [6]. Here, we investigate whether human neurons are capable of using spider silk as guiding scaffold for neurite growth. We show that human model neurons attach to the silk fibers which provide a permissive and nonimmunogenic biomaterial for neurite growth. Since more than half of the cultured NT2 neurons have a cellular phenotype resembling central neurons, the spider silk fibers are an excellent candidate biomaterial for enhancing neural repair in the injured central nervous system.

## 2. Material and Methods

### 2.1. Animal Handling

Spiders of the species* Nephila* sp. were held in the rooms of the animal facility of the Department of Plastic, Hand, and Reconstructive Surgery. Animals were fed three times a week with decapitated crickets and their webs were humidified daily. For silk harvesting only female individuals with an age of 3 to 12 months were used. Spiders were taken back to their web and were fed and humidified independently of the feeding interval after silk was harvested.

### 2.2. Rearing of the Silk

In accordance with the German Animal Welfare Law and the European Directives, no allowances for handling and treatment were needed. Stress level for animals was kept to an absolute minimum and no harming occurred during the harvesting process. For this purpose spiders were fixed with gauze and needles to a styropor pad without anesthesia as already described [7]. For dragline silk collection, the silk was pulled out of the major ampullate gland with forceps. The mechanical stimulus is sufficient to start silk production. The end of the dragline was mounted to a motorized drum and the harvesting lasted until the animal became anxious as already described by Kuhbier and colleagues [8].

### 2.3. Scaffold Preparation

Medicinal stainless dental steel frames (diameter of 0.7 mm, Dentaurum, Ispringen, Germany) were bent to rectangular frames with edge lengths of approximately 5 mm as described in former studies [8]. Dragline spider silk was woven around the frames with 20 to 30 fibers in parallel orientation. Woven frames were autoclaved for 2 hours after weaving and kept sterile until use in cell culture experiments. Round glass cover slips with a diameter of 25 mm (Glaswarenfabrik Karl Hecht KG, Sondheim, Germany) were cleaned with 70% ethanol and put under a binocular loupe. Briefly, single silk fibers were oriented parallel with a distance of 400 to 800 *μ*m. Fibers were fixated on the cover slip with either nail polish, superglue, Sylgard 185 elastomer (Sigma-Aldrich, Taufkirchen, Germany), or Surgibond tissue adhesive (SMI, St. Vith, Belgium). Scaffolds were sterilized by steam autoclaving or UV radiation under the clean bench for at least 90 minutes.

### 2.4. Neuronal Differentiation

Human NT2/D1 precursor cells (NT2) were purchased from the American Type Culture Collection (ATCC, Manassas, VA, USA). Neuronal precursors were treated as described earlier by Paquet-Durand et al. [9] and Podrygajlo et al. [10]. When precursors reached confluence on T175 culture flasks (Sigma-Aldrich), cells were washed two times and trypsinized (Trypsin-EDTA, Gibco-Invitrogen, Karlsruhe, Germany). For neuronal differentiation, 5 × 10^6^ precursor cells were transferred to a bacteriological grade Petri dish (Greiner, Hamburg, Germany) with 10 mL Dulbecco's modified Eagle medium (DMEM/F12) supplemented with 10% fetal bovine serum, 1% penicillin and streptomycin, and 10 *μ*M retinoic acid. Cells were cultured for 7–10 days with medium being changed three times a week. Approximately 4 × 10^6^ cell was then transferred to a T75 culture flask (Greiner) with DMEM/F12 medium with penicillin/streptomycin and retinoic acid. Ten days later, with medium being changed three times a week, the neuronal precursors were washed two times with PBS (phosphate buffered saline) and trypsinized. Cells were cultured for 48 hours with DMEM/F12 without retinoic acid in a T175 culture flask at a density of 1 × 10^8^ cells/flask. Two days later, cells were washed, trypsinized, and transferred to T75 culture flasks with DMEM/F12 inhibitor medium containing 5% fetal bovine serum, 1% penicillin/streptomycin, and mitotic inhibitors (1 *μ*M 1-6-D-arabinofuranosylcytosine, 10 *μ*M 2′-deoxy-5-fluorouridine, and 10 *μ*M 1-*β*-D-ribofuranosyluracil). After neurons became visible under inhibitor treatment (7–12 days), neurons were washed with PBS and selectively trypsinized. Human NT2 neurons were counted and used in the experiments on steel frames and cover slips. All experiments were performed with neurons of passage 20–30.

### 2.5. Immunocytochemistry

Stainings were performed as described earlier by Podrygajlo et al. [10] and Tegenge et al. [11]. Neurons were rinsed with PBS and then fixed with 4% paraformaldehyde (PFA) for 15 minutes at room temperature. Cells were washed three times with PBS containing 0.1% Triton X-100 (PBST) to remove the remaining PFA. Unspecific binding site was blocked with 5% normal horse serum in PBST for at least one hour at room temperature. For visualizing neurons, cells were stained with monoclonal antibody *β*-III-tubulin (1 : 10.000, Sigma-Aldrich) diluted in block solution over night at 4°C. After three wash steps with PBST, biotinylated secondary antibody horse-against-mouse (1 : 250, Vector, Burlingame, MA, USA) in PBST was applied for 60 minutes at room temperature. Before incubation with streptavidin-coupled Cy3 (1 : 250, Sigma-Aldrich) and DAPI (0.1 *μ*g/mL, Sigma-Aldrich) in PBST, three wash steps with PBST were carried out.

### 2.6. Measurement of Neuronal Aggregation

To quantify aggregation of human model neurons (NT2) on spider silk, we measured the area covered by DAPI stained neuronal nuclei in three stripes that were aligned in parallel to silk fibers. The rectangular size of stripes was chosen as 40 *μ*m × 330 *μ*m. The first stripe covered the area of the silk fiber and approximately ±20 *μ*m to the left and right. The second and the next adjacent stripes ran in parallel to the first stripe, such that the outermost stripe was in a distance of more than 50 *μ*m away from the spider silk. This outermost stripe was considered as control region which allowed for neurite growth without silk fiber influence. The DAPI covered area was measured using ImageJ 1.47 (http://rsbweb.nih.gov/ij/). Using the free hand selection tool, we encircled each neuronal nucleus. We calculated the DAPI covered area by subtracting the summed area of all nuclei from the total stripe area.

## 3. Results and Discussion

**Figure 1:**
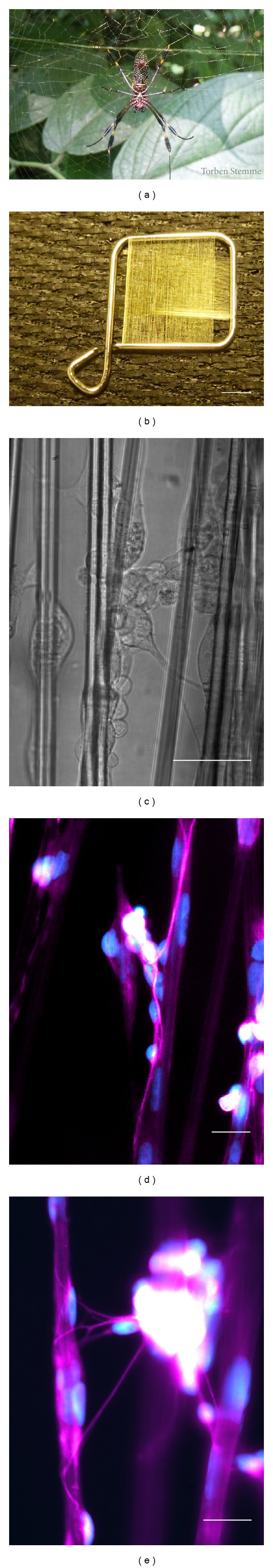
Human model neurons growing on steel frame wired spider silk fibers of* Nephila* sp. (a) Image of a female individual of the species* Nephila* sp. (b) Handcrafted stainless dental steel frame with cross-woven spider silk. (c) Phase contrast image of human NT2 neurons attached to fibers of the spider silk. ((d)-(e)) Neurons stained against neuronal *β*-type-III-tubulin (magenta) and DAPI (cyan) with neurites growing on the fibers and neurons bridging the gap and making contact to other fibers. Scale bars are 0.25 mm (b) and 25 *μ*m ((c)–(e)).

**Figure 2:**
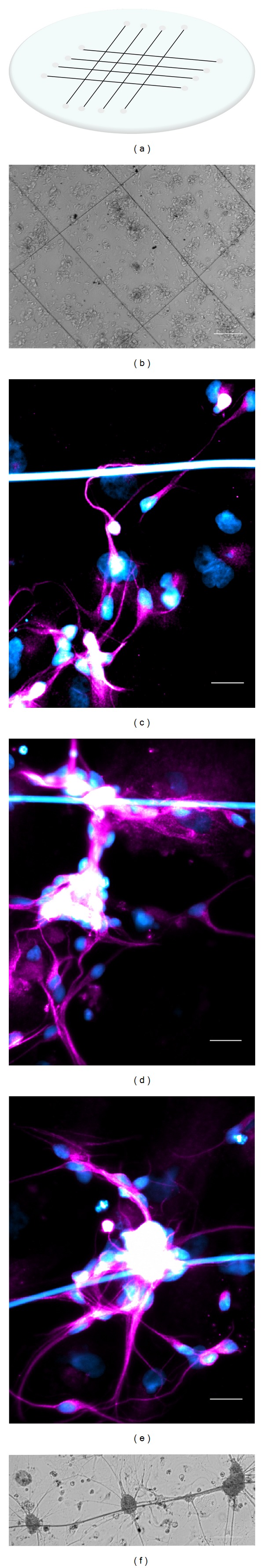
Cross-fixed spider silk fibers on cover slips with human model neurons forming ganglion-like structures. (a) Scheme of a 25 mm glass cover slip with 4 × 4 cross-fixed silk fibers. (b) Phase contrast image of NT2 neurons seeded on a cover slip with spider silk fibers fixed to it. (c) Neurons establishing initial contact with a fiber of spider silk after 24 hours in culture. The outgrowing neurite uses the silk fiber as guidance scaffold. (d) After 36 hours, more neurons established contact to the silk fiber and started relocation of the soma from glass surface to the silk fiber. (e) After 5 days in culture, aggregates formed by neurons became bigger with bundles of neurites emerging into the surrounding area. (f) After three to four weeks in culture, neurons seeded to spider silk fibers formed large ganglion-like structures along the silk. Neurons are stained against neuronal *β*-type-III-tubulin (magenta) and DAPI (cyan) to visualize the nuclei. Scale bars are 200 *μ*m ((b), (f)) and 25 *μ*m ((c)–(e)).

Initially we used silk fibers wrapped around a steel frame. Preliminary experiments have shown successful adhesion of cells to spider silk woven steel frames* in vitro* (Figure 1). Dispersed neurons were transferred in a small amount of cell culture medium and placed on top of the cross wove spider silk of the species* Nephila* sp. (Figures 1(a)-1(b)). Within two hours after seeding, neurons were attached to the silk, whereas nonadhering neurons dropped to the bottom of the culture plate. Light microscopy phase contrast images revealed small aggregates of cell soma and neurites crossing the gap between single silk fibers after several hours of culturing (Figure 1(c)). Within 24 h after seeding of neurons, small neurites arising from the silk attached neurons extended along the fibers, using them as guidance structures and outgrowth substrate (Figure 1(d)). Neurons forming small aggregates send their neurites not only along the silk fibers, but also spanned the 50 *μ*m gap between single silk fibers and connected to an adjacent silk fiber (Figure 1(e)). Neurites not only attached to the silk fiber but also bifurcated, formed small hooks, and grew spirally around the fiber to maximize contact area with the silk. However, measuring of neurite lengths and monitoring of single cells in a live imaging apparatus were difficult to obtain. The silk fibers woven around the steel frames are not in the same focal plane and tracing of single neurites or even growth cones was impractical. To improve optical imaging, we used a different approach of silk mounting. We briefly fixed single fibers of the dragline silk using nail polish, superglue, Sylgard 185 elastomer, or a tissue adhesive to glass cover slips. Silk fibers were oriented as 4 fibers parallel and 4 additional fibers rotated 90° to them (Figures 2(a) and 2(b)). Fixation of silk fibers with nail polish and superglue showed satisfactory results until steam autoclaving and UV sterilization. During immunocytochemistry staining procedure, small spots of glue and polish fixating the spider silk disintegrated and the silk meshwork detached from the glass cover slip. The use of Sylgard and the tissue adhesive Surgibond abolished these problems. Eventually, we decided to use UV sterilization as standard procedure.

### 3.1. Crossed Fiber Array

**Figure 3:**
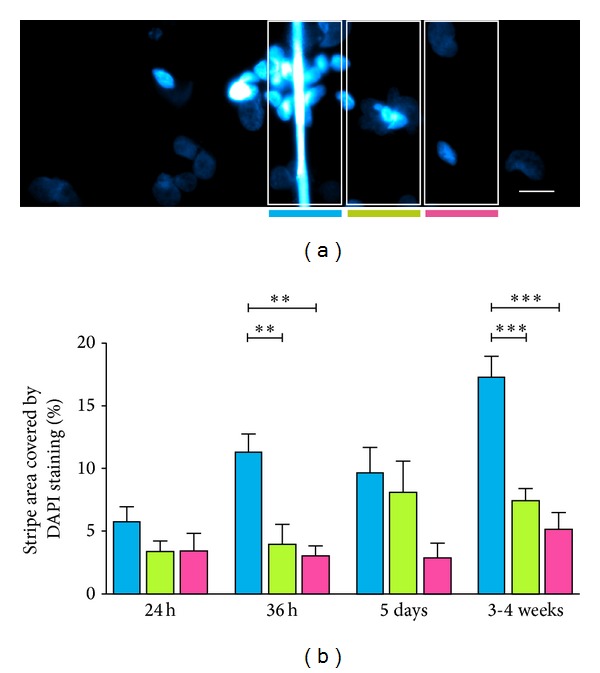
Human model neurons aggregating on the spider silk over culture time. (a) Detailed view of an image used to quantify the rate of aggregation of neurons. Three stripes parallel to a spider silk fiber were evaluated by measuring the area covered with DAPI staining, representative of the amount of neuronal nuclei. (b) Within 24 hours after seeding neurons to crossed fiber arrays, no significant difference in DAPI covered area between the stripes could be detected. After 36 hours significantly more neuronal nuclei could be seen in the stripe covering the silk fiber than in the adjacent stripe, or the most distant stripe to the silk. After 3-4 weeks in culture the difference between the stripe covering the silk and the outer stripes is highly significant (∗∗∗). Data are presented as mean and SEM. Significance levels are **P < 0.01 and ***P < 0.001 using one-way ANOVA and Bonferroni post hoc test. Nuclei of neurons are stained with DAPI (cyan). Scale bar is 20 *μ*m.

To assemble for better microscopy and arrangement of the silk in a focal plane, fibers were glued in a crossed fiber pattern (Figure 2(a)) to a glass cover slip. Adhesion of cells was most effective in this crossed fiber array while preserving the adhesiveness to the silk material shown in the parallel arrangement. After 24 hours, neurons established initial contact with single silk fibers and used it as guidance structure for the elongating neurite (Figure 2(c)). After 36 hours, additional neurons touched the silk and oriented their cell bodies along the fibers (Figure 2(d)). Neurons in the surrounding area of silk fibers extended their neurites and contacted neurons already attached to the silk. After 5 days in culture, the ganglion-like cell clusters on the spider silk increased in size. Neurons of these clusters extended their neurites in the surrounding area and stayed in contact with neurons not part of the ganglion-like aggregates. After 3-4 weeks of culturing human neurons on single fibers of spider silk, ganglion-like structures became even larger and formed thick bundles of neurites along the fibers to establish contact to other ganglion-like structures. Other neurons of the aggregates formed bundles of neurites stretching into the surrounding area. To quantify the aggregation process of the neuronal cell bodies on spider silk over culture time, we measured the area covered by DAPI positive nuclei in three parallel stripes along the silk fibers. These measurements revealed significant differences in neuronal attachment to silk after 36 hours and after several weeks in culture (Figure 3(b)). After 36 hours 11% of a stripe along the silk was covered with neuronal nuclei. The areas covered in a stripe adjacent to or more than 50 *μ*m away from the silk were 4% and 3%, respectively. After 3 to 4 weeks in culture, DAPI covered area in the silk stripe was above 17%, whereas the adjacent and distant stripes had covered areas of 7.4% and 5%. Since the terminally differentiated neurons do not increase cell numbers anymore in culture [12], these results show that outgrowing neurites attached to the silk fibers mechanically pull the somata away from the bottom of the cover slip towards the preferred substrate of silk, where they assemble in cellular aggregates. The movement of neuronal cell bodies towards the silk fiber may also, to some extent, explain the result after 5 days in culture (Figure 3(b)), where no significant difference was found in the statistical treatment, but the area of neuronal nuclei in the second stripe seemed to have increased. Several studies have investigated the attachment of nonneuronal cell types to spider silk. Adhesion and proliferation of 3T3 fibroblasts seeded to spider silk woven steel frames were reported earlier by Kuhbier and colleagues [8]. A work of Widhe and colleagues has shown that adhesion of human fibroblast to matrixes of the recombinant miniature spider silk protein 4RepCis was successful using foam, fibers and mesh [13]. Best rate of binding was, however, reported for a combination of film, mesh, and fibers. Fibroblast was attached to the silk protein and showed an elongated shape following the orientation of the fibers. Neurons grown in aligned fibers of peptide amphiphile (PA) scaffolds showed a directed growth of P19 neurons [14]. Dorsal root ganglion cells embedded into PA scaffold showed extensive migration over hundreds of micrometers within days. In contrast to spider silk scaffold, it is necessary to embed the cells into the peptide amphiphile, being a major disadvantage over spider silk. Bern and colleagues reported extensive neurite outgrowth and migration within the PA scaffold, but no migration in the surrounding area was observed [14]. However, we show here for the very first time that CNS human neurons seeded to spider silk fibers on steel frames as well as to the crossed fiber arrays resulted in outgrowth of neurites and orientation of the cell bodies along the silk fibers. A similar outgrowth pattern with neurons aligned to the silk fibers was reported earlier after transplantation of spider silk scaffolds into a 20 mm sciatic nerve defect in rats [15]. Transplantation of nerve constructs consisting of decellularized venules and a thick bundle of spider silk into a tibial nerve defect in sheep resulted in axonal regeneration [6]. Regenerated neurons had elongated their neurites throughout the transplanted scaffold and bridged a defect distance of approximately 6.0 cm. Similar attempts are made using silk conduits of* Bombyx mori* containing Spidrex to improve regeneration of peripheral neurons in the sciatic nerve of rats [16]. Assessment* in vitro* showed that Spidrex supported neurite elongation of rat primary dorsal root ganglion cells. Transplantation in the rat tibial nerve resulted in neuronal regeneration similar to autologous nerve graft transplantation [16]. We further showed that human CNS neurons formed ganglion-like structures when cultured for several days on crossed fiber arrays. This tendency of process bearing NT2 neurons to form cell clusters has already been shown for 3-4-week-old dissociated cell cultures [9, 10, 17]. The position of the cell clusters showed an even distribution over the whole culture plate, whereas ganglion-like aggregates in crossed fiber arrays are predominantly associated with silk fibers. Formation of ganglion-like structures was earlier reported for hippocampal neurons and astrocytes cultured on porous aragonite skeleton obtained from biofabricated hydrozoan [18]. These ganglion-like spheres were attached directly to the surface of the scaffold or via a neck of cells. Here, we show similar ganglion-like structures formed on biodegradable silk from* Nephila* sp. Based on the percentage of GABAergic (15%) and glutamatergic (40%) cells using amino acids as likely transmitters [9], we can assume that more than 50% of the differentiated neurons in cell culture do not reflect the motoneuronal phenotype but rather correspond to central neurons. This would allow for the investigation of neuronal repair mechanisms and enhanced regeneration of central axonal connectivity in human NT2 neurons attached to the spider silk fibers.

## 4. Conclusions

This is the first report about human model neurons attaching to spider silk fibers woven on steel frames. We further developed a new technique to monitor neurite growth along single fibers. Using the crossed fiber arrays, we showed that neuronal cell bodies contacted the silk fibers and formed ganglion-like structures within 4 weeks. This new technique offers the possibilities to optimize the properties of spider silk for enhanced neurite regeneration before transplantation of the biomaterial into injured human nerve tissue.
